# Characteristics of Spontaneous Square-Wave Jerks in the Healthy Macaque Monkey during Visual Fixation

**DOI:** 10.1371/journal.pone.0126485

**Published:** 2015-06-11

**Authors:** Francisco M. Costela, Jorge Otero-Millan, Michael B. McCamy, Stephen L. Macknik, Leandro L. Di Stasi, Héctor Rieiro, John R. Leigh, Xoana G. Troncoso, Ali Najafian Jazi, Susana Martinez-Conde

**Affiliations:** 1 Department of Neurobiology, Barrow Neurological Institute, Phoenix, AZ, United States of America; 2 Interdisciplinary Graduate program in Neuroscience, Arizona State University, Tempe, AZ, United States of America; 3 Department of Neurology, Johns Hopkins University, Baltimore, MD, United States of America; 4 Department of Ophthalmology, State University of New York Downstate Medical Center, Brooklyn, NY, United States of America; 5 Mind, Brain and Behavior Research Center; University of Granada, Granada, Spain; 6 Department of Signal Theory and Communications, University of Vigo, Vigo, Spain; 7 Veterans Affairs Medical Center, Case Western Reserve University, Cleveland, OH, United States of America; 8 Unité de Neuroscience, Information et Complexité (CNRS-UNIC), UPR CNRS 3293, Gif-sur-Yvette, France; UMR8194, FRANCE

## Abstract

Saccadic intrusions (SIs), predominantly horizontal saccades that interrupt accurate fixation, include square-wave jerks (SWJs; the most common type of SI), which consist of an initial saccade away from the fixation target followed, after a short delay, by a return saccade that brings the eye back onto target. SWJs are present in most human subjects, but are prominent by their increased frequency and size in certain parkinsonian disorders and in recessive, hereditary spinocerebellar ataxias. SWJs have been also documented in monkeys with tectal and cerebellar etiologies, but no studies to date have investigated the occurrence of SWJs in healthy nonhuman primates. Here we set out to determine the characteristics of SWJs in healthy rhesus macaques (Macaca mulatta) during attempted fixation of a small visual target. Our results indicate that SWJs are common in healthy nonhuman primates. We moreover found primate SWJs to share many characteristics with human SWJs, including the relationship between the size of a saccade and its likelihood to be part of a SWJ. One main discrepancy between monkey and human SWJs was that monkey SWJs tended to be more vertical than horizontal, whereas human SWJs have a strong horizontal preference. Yet, our combined data indicate that primate and human SWJs play a similar role in fixation correction, suggesting that they share a comparable coupling mechanism at the oculomotor generation level. These findings constrain the potential brain areas and mechanisms underlying the generation of fixational saccades in human and nonhuman primates.

## Introduction

**Fig 1:**
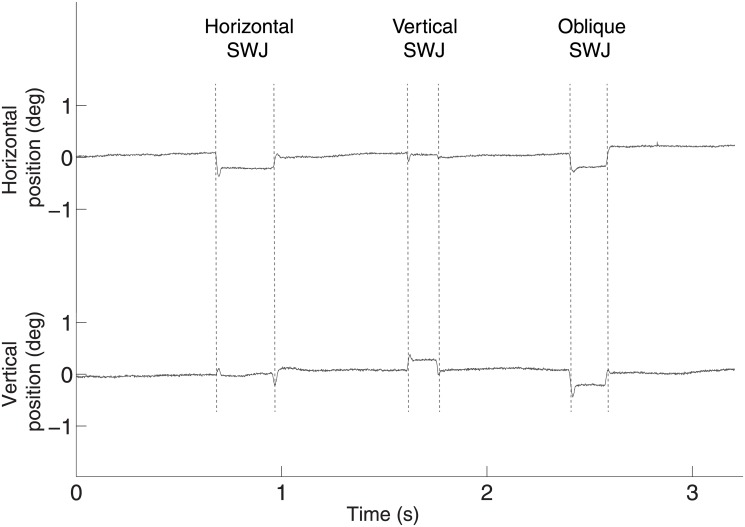
Examples of primate SWJs. Each trace represents 3 seconds of horizontal (top) and vertical (bottom) eye position recordings containing SWJs. Primarily horizontal (left), vertical (middle), and oblique (right) SWJs are displayed. All time scales are as in the bottom trace.

Attempted visual fixation of a target is repetitively interrupted by saccadic intrusions (SIs) and fixational eye movements. Square-wave jerks (SWJs), the most frequent type of saccadic intrusion (SI), consist of an initial saccade away from the fixation target, followed by a return saccade that brings the eye back onto target (Fig 1) [1].

SWJs are present in most human subjects, but occur with increased frequency in patients with progressive supranuclear palsy (PSP), Parkinson's disease, and recessive, hereditary spinocerebellar ataxia [1–6].

Fixational eye movements include tremor, drift, and microsaccades (i.e. small-magnitude saccadic eye movements, also called fixational saccades) [7–9] and can be observed in human and nonhuman primates, as well as in other foveate species [10].

Recent research has shown that SWJs and microsaccades share similar features in both healthy human subjects and in patients with a variety of neurological disorders [4,5], and suggests that SIs (including SWJs) and microsaccades are part of a continuum of fixational instabilities [11], generated by a common neural circuit [8].

Previous studies have moreover reported irrepressible saccades and SWJs in monkeys with tectal and cerebellar lesions [12–14]. It is not known, however, if SWJs are also present in healthy nonhuman primates. This question is interesting, not only because of the importance of the macaque monkey brain to oculomotor neurophysiology studies, but also because, unlike human microsaccades and saccadic intrusions, which are predominantly horizontal, microsaccades in monkeys do not typically display a horizontal preference [15]. Here we set out to determine the characteristics of SWJs in healthy rhesus macaques during attempted fixation of a small visual target.

From here on, for simplicity, we will refer to all (micro)saccades made during attempted fixation as fixational saccades or, simply, saccades, regardless of size.

## Methods

### 3.1. Animals

Eye position was recorded monocularly at 1000 Hz with a scleral search coil [16–18] Recordings included data from five awake rhesus macaques (Macaca mulatta). Three monkeys were studied at Harvard Medical School (eye-tracking equipment by Remmel Labs, Inc) and two monkeys were studied at the Barrow Neurological Institute (eye-tracking equipment by Riverbend Instruments, Inc). Standard sterile surgical techniques, recording procedures, and animal care methods were approved by the Harvard Medical School Standing Committee on Animals and the Institutional Animal Care and Use Committee at Barrow Neurological Institute. Monkeys fixated their gaze on a small fixation target (0.5 degrees of visual angle [deg], with a luminance of 24.3 cd per m^2^) on a video monitor (Reference Calibrator V, 60–120 Hz refresh rate; Barco) placed at a distance of 57 cm. Fruit juice rewards were provided for every ~1.5–2 seconds of fixation. Eye movements exceeding a 2 x 2 deg fixation window were recorded but not rewarded. Three of the monkeys were tested during previously reported studies that addressed different experimental questions [17–19].

The animals were bred in captivity and housed individually in nonhuman primate cages (group 4; dimensions 89 cm width, 147 cm height, 125 cm depth, including perch) for the duration of the experiment. Monkeys were provided with several kinds of environmental enrichment, including a television, various fruits and vegetables, food puzzles, perches, Kong toys, mirrors, and other enrichment tools as available, along with visual and auditory contact with several other monkeys that were also housed individually in the same room, and positive daily human contact. The room had a 12 hour light/dark cycle. Regular veterinary care and monitoring, balanced nutrition, and sensory and social environmental enrichment were provided in accordance to the National Institutes of Health Guide for the Care and Use of Laboratory Animals and the EU Directive 2010/63/EU for animal experiments, to maximize physical and psychological well-being. Monkeys had abundant access to food (i.e. feed biscuits were provided twice a day (approximately 12 biscuits/monkey), Purina Lab Diet Monkey Diet, Product# 0001333). Daily fluid intake was controlled and monitored during the experiments. Monkeys typically earned over 80% of their daily fluid allotment during the testing sessions, and received water and/or fruit supplements after the experiments. Whenever the animals were not actively participating in testing or training sessions (i.e. weekends, data analysis and manuscript writing periods, etc.), they had free access to water in the vivarium.

Cranial head-post and scleral search-coil implantation surgeries were conducted previous to the eye movement recordings, under general anesthesia using aseptic techniques, and with full post-operative analgesia and antibiotic therapy. No animals were sacrificed at the end of the experiments. We followed the ARRIVE (Animal Research: Reporting of In Vivo Experiments) guidelines and the ARRIVE Checklist is available as supporting information (S1 Checklist).

### 3.2. Eye movement analyses

#### 3.2.1 Saccade Detection

**Fig 2:**
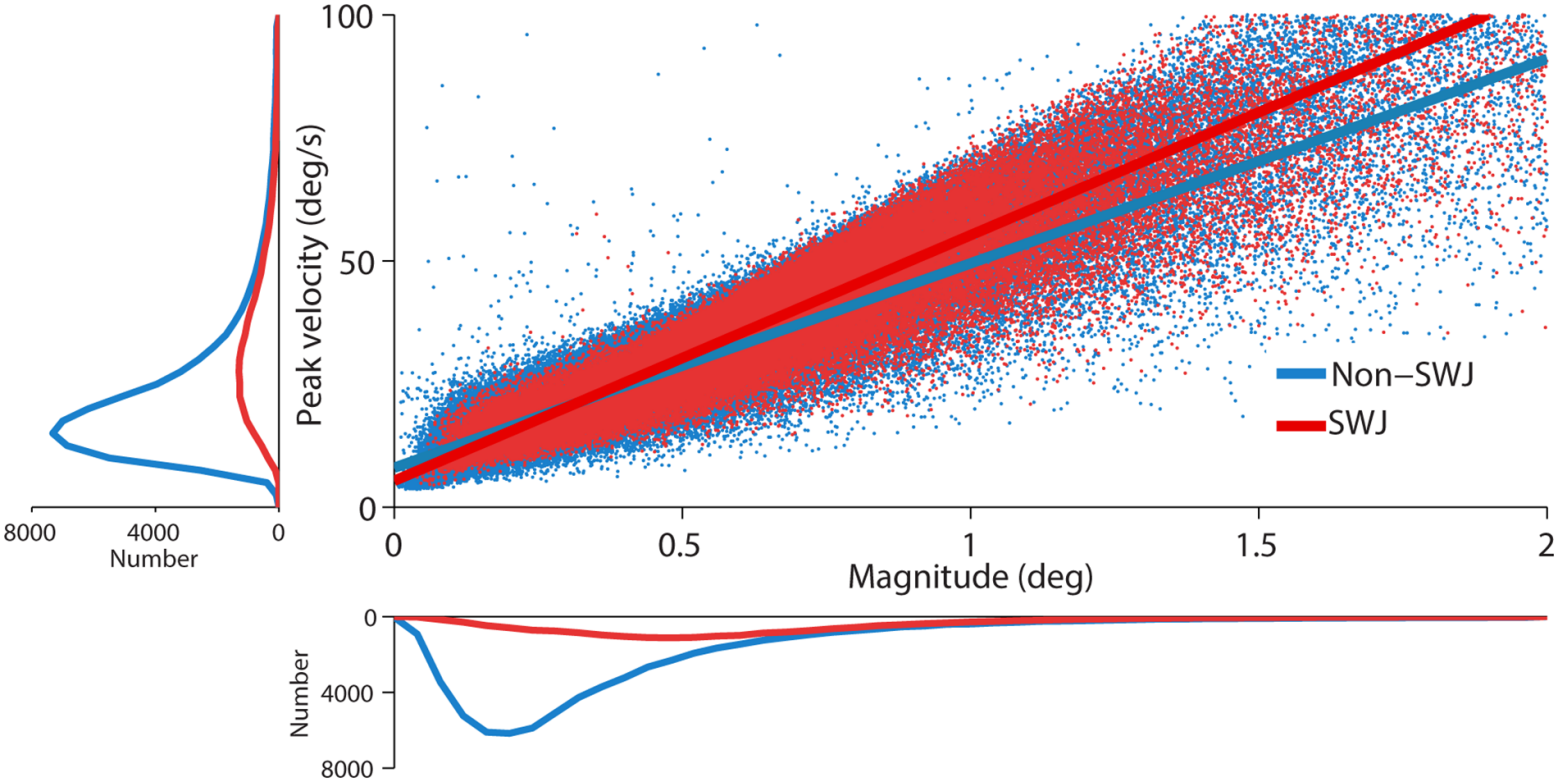
Peak velocity-magnitude relationship for SWJ saccades and non-SWJ saccades. Main panel: Each dot represents a saccade with the peak velocity indicated on the y-axis and the magnitude on the x-axis. Color determines whether the saccade was part of a SWJ (red) or not (blue). Bottom panel: Average saccade magnitude distribution across monkeys (*n* = 5). Left panel: Average peak velocity distribution across monkeys (*n* = 5). Saccade magnitude and peak velocity were greater for SWJ saccades than for non-SWJ saccades (Z-values = 2.02; *p*-values = 0.04). The slopes of the peak velocity-magnitude relationships for SWJ saccades and non-SWJ did not differ statistically (Z-value = 0.14; *p* = 0.89).

We identified all saccadic eye movements automatically with a modified version of the algorithm developed by Engbert & Kliegl [20–24]. This method detects saccades in the two-dimensional velocity space using a threshold that adapts to the level of noise of each recording. We set λ = 8 (used to obtain the velocity threshold) and established a minimum saccadic duration of 8 msec. Some saccades are followed by a fast and small saccadic eye movement in the opposite direction, called dynamic overshoot, which is often more prominent in the eye that moves in the abducting direction [25]. Unlike the return saccade in a SWJ, a dynamic overshoot follows a saccade without latency between the two movements. We identified dynamic overshoots as saccades that occurred less than 20 ms after a preceding saccade [26–29], and considered them part of the preceding saccade (i.e. we did not regard them as new saccades). That is, we discarded the second saccade and modified the end point of the first saccade to include the overshoot. Fig 2 shows the peak velocity-magnitude relationship (main sequence) for fixational saccades with magnitudes < 2 deg [6,30–34], and the corresponding saccade magnitude and peak velocity distributions (see S2 Fig for the saccade magnitude distributions for each individual monkey).

#### 3.2.2 SWJ Detection

We defined a SWJ as the combination of one small saccade that moves the eye away from the fixation target, followed after a short period by a second corrective saccade directed back towards the target [1,4,35,36] (Fig 1). To characterize SWJs in an objective manner, we first identified all individual saccades up to 5 deg [4]. We chose this 5-deg upper magnitude threshold to include the range of SWJ magnitudes reported previously in healthy human subjects (0.1–4.1 deg; [1]), as well as in neurological patients [4].

We identified SWJs by measuring how similar a given saccade pair (that is, a pair of consecutive saccades) was to an ideal SWJ. In an “ideal SWJ” the two saccades are separated by a short interval (usually around 200 ms), have the same magnitudes, and their directions are exactly opposite [4–6]. We calculated a SWJ index based on these three defining SWJ characteristics: (a) the direction dissimilarity of first and second saccade, (b) the magnitude similarity of first and second saccade, and (c) the temporal proximity of first and second saccade. The SWJ index provides a single, continuous variable between zero and one for each saccade pair. Values closer to one indicate more similarity to an ideal SWJ. If a saccade pair’s SWJ index was larger than a given threshold (0.0014; the same index as in our previous SWJ studies, see [4,6]), we classified the pair as a SWJ. See S3 Fig for the distribution of SWJ indices. By "SWJ saccades", we refer to the two saccades that define a complete SWJ. By "non-SWJ saccades", we refer to any other saccades that are not part of SWJs, including fixational saccades. We defined SWJ magnitude as the average magnitude of the two saccades defining the SWJ. We defined likelihood of being part of a SWJ as the percentage of saccades from the pool that were considered as part of SWJ, detected by our algorithm.

### 3.3. Statistical methods

To compare the characteristics of saccades inside and outside SWJs, we performed separate Wilcoxon’s signed-rank tests for each dependent variable (saccadic magnitude, peak velocity, slope of the peak velocity/magnitude relationship, direction, vertical component, and polar asymmetry). To calculate the vertical component of saccades of different magnitudes, we first normalized the magnitudes of all saccades to 1 deg. Polar asymmetry was defined as $\text{A}=\sqrt{\sum_{\theta }^{\frac{\pi }{\text{2}}}{\left(\text{H}\left(\theta \right)-\text{H}\left(\theta +\frac{\pi }{2}\right)\right)}^{2}}$, where A is the Euclidean distance between a given point in the polar histogram and its symmetric counterpart. To assess the relationship between saccadic magnitude and the likelihood of being part of a SWJ, we performed separate Friedman’s tests for the saccadic vertical component, likelihood of being part of a SWJ, and intra-SWJ inter-saccadic interval [ISI]. Saccadic magnitude was the within subjects factor variable (10 bins of 0.2 deg each). We also considered the distance to fixation target as a within subjects factor (10 bins of 0.2 deg each) for the likelihood of being part of a SWJ and intra-SWJ ISI. Finally, we ran logistic regressions between a saccade’s likelihood of being part of a SWJ, and the saccadic magnitude and post-saccadic distance to the fixation target, considering the saccade pool from all monkeys. Significance levels were set at *p* < 0.05 throughout.

## Results

SWJs are the most common type of SI in human subjects. Here we set out to determine the characteristics of spontaneous SWJs in five healthy rhesus macaques during attempted visual fixation of a small target. Eye movements recorded during the fixation task consisted primarily of fixational saccades (i.e. microsaccades) and drifts, in addition to occasional larger saccades and spontaneous blinks.

### 4.1 Characteristics of SWJ saccades and non-SWJ saccades

All five primates produced spontaneous SWJs. The average likelihood for any given saccade to be part of a SWJ was 17.85% (SD +/-19%) (Fig 1), a slightly lower value than previously reported for healthy human subjects [4,6].

Consistent with prior human studies [4,6], saccades being part of SWJs (heretofore SWJ saccades) had greater magnitudes and peak velocities than saccades not being part of SWJs (heretofore non-SWJ saccades). The peak velocity-magnitude relationships were comparable for SWJ saccades and non-SWJ saccades, and their corresponding linear fit slopes did not differ statistically (Fig 2, Table 1).

**Table 1:** Characteristics of SWJs and non-SWJ saccades.

	SWJ saccades	non-SWJ saccades
**Rate (N/s)**	0.27 (0.38)	1.08 (0.30)
**Magnitude (deg)** *	0.50 (0.19)	0.39 (0.12)
**Peak velocity (deg/s)** *	28.93 (9.23)	23.60 (6.20)
**Saccadic slope (deg/s)**	48.37 (3.73)	41.95 (5.66)
**Vertical component**	0.57 (0.21)	0.56 (0.26)
**Polar asymmetry** *	2.75 (1.17)	7.01 (2.94)

Medians and SDs are indicated for each variable and calculated across monkeys (*n* = 5).

* indicates *p* < 0.05, Wilcoxon’s signed-rank test.

**Fig 3:**
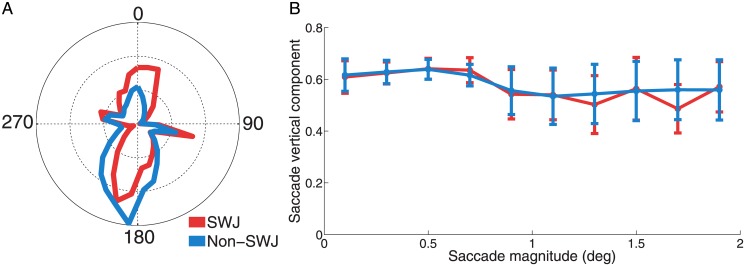
Direction of SWJ saccades and non-SWJ saccades. **A) Polar histogram of saccade directions for SWJ saccades and non-SWJ saccades.** We show the average saccade direction distribution across monkeys (*n* = 5). Both SWJ saccades and non-SWJ saccades are biased vertically. Whereas downward-directed component is prominent for both SWJ saccades and non-SWJ saccades, the upward-directed component is more pronounced for SWJ saccades than non-SWJ saccades. Median Euclidean distance values were significantly lower for SWJ saccades than for non-SWJ saccades, indicating higher symmetry (Z-value = 2.02; *p* = 0.04; see Methods). **B) Saccade magnitude and vertical component.** The vertical component remained constant across saccadic magnitudes for both SWJ saccades (Friedman's test (5, 9) = 5.50; *p* = 0.787) and non-SWJ saccades (Friedman's test (5, 9) = 3.19; *p* = 0.956). Error bars represent the s.e.m. across monkeys (*n* = 5).

Human SWJs are typically composed of horizontal saccades, both in neurological patients and in healthy individuals [1,4,5]. Here we found that such horizontal preference does not extend to SWJs in the macaque monkey, as both SWJ saccades and non-SWJ saccades had a strong vertical component (Fig 3A and S1 Fig). Polar asymmetry was larger for non-SWJ saccades than for SWJ saccades, however, consistent with the square-wave coupling that characterizes SWJs, where the second (i.e. return) saccade has roughly opposite direction to that of the first saccade (Fig 3A, Table 1).

In human subjects, the fixational saccadic preference for horizontal direction decreases moderately with saccade magnitude (Otero-Millan et al., 2011b). In contrast, the primate fixational saccadic preference for vertical direction [15] was unrelated to saccade magnitude, either for SWJ saccades or non-SWJ saccades (Fig 3B, Table 1).

### 4.2 SWJs and fixation correction

**Fig 4:**
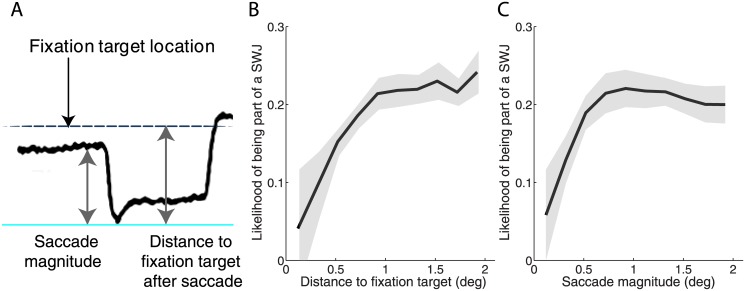
Relationship between fixation error and subsequent SWJ generation. **A) Schematic illustration of a fixation error.** The fixation target location, the magnitude of the first saccade in the SWJ, and the distance to the fixation target after the first SWJ saccade are indicated. Relationship between a saccade’s likelihood of being part of a SWJ and **B) the post-saccadic distance to the fixation target and C) the saccade’s magnitude.** Both relationships follow a logistic regression (*p*-values < 0.05), where a saccade’s likelihood of being part of a SWJ increases with both the distance to the fixation target after the saccade [Friedman's test (5, 4) = 12.32; *p* = 0.015] and the saccadic magnitude [Friedman's test (5, 4) = 16.64; *p* = 0.002)] up to 1 deg, and then plateaus for values > 1 deg [*p*-values >0.9]. Grey shadows indicate the s.e.m. across monkeys (*n* = 5).

As human fixational saccades increase in size, they are more likely to be followed by a return saccade, indicating a role of SWJs in fixation correction (Otero-Millan et al., 2011b). To determine if primate SWJs might be similarly corrective, we examined the relationship between the presence of a fixation error and the likelihood of square-wave coupling. We considered the following two measures of fixation error: a) saccadic magnitude (Fig 4A, first gray arrow from the left), and b) the distance between post-saccadic gaze position and fixation target location (Fig 4A, second gray arrow from the left). We found that SWJ likelihood increased as a function of the size of the fixation error: The larger the distance between the post-saccadic gaze position and the fixation target location, the more likely the trigger of a return saccade (Fig 4B). Likewise, large saccades were more likely than small saccades to lead to square-wave coupling in the form of return saccades (Fig 4C).

We note that the distribution of distances from the end of the second saccade to the fixation target (calculated as the median eye position per session), and from the end of the second saccade to the beginning of the first saccade (i.e. the beginning of the SWJ) did not differ significantly (S4 Fig). Thus, the present data cannot elucidate whether the second SWJ saccades are scaled to reach the fixation target or go back to the pre-first saccade position.

**Fig 5:**
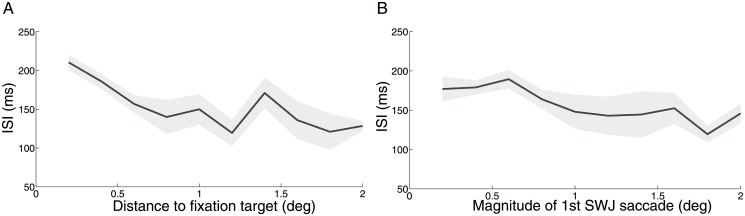
Relationship between fixation error and Intra-SWJ ISI. A) The larger the distance to fixation target, the shorter the intra-SWJ ISI [Friedman's test (5, 9) = 17.55; *p* = 0.047)]. B) Likewise, larger saccades tend to be more quickly followed by return saccades [Friedman's test (5, 9) = 18.80; *p* = 0.026)]. Shadows indicate the s.e.m. across monkeys (*n* = 5).

We also found that the larger the fixation error (defined as a function of either saccade magnitude or post-saccadic distance to the fixation target), the shorter the time lapsed from the end of the first SWJ saccade to the beginning of the second SWJ saccade (Intra-SWJ inter-saccadic interval; ISI; median: 180.60 ms SD +/- 23.54) (Fig 5).

## Discussion

We set out to investigate the occurrence, and determine the characteristics, of SWJs in the healthy, awake fixating primate. Prior studies found an abundance of SWJs, not only in neurological patients, but also in healthy human subjects [1,4–6]. SWJs have also been reported in monkeys suffering from tectal and cerebellar lesions [12–14], but it remains unknown whether healthy nonhuman primates present SWJs during attempted visual fixation, and if so, whether the characteristics of primate SWJs are comparable to those of human SWJs.

Here we set out to determine the characteristics of spontaneous SWJs during attempted fixation in five healthy rhesus monkeys, and found SWJ occurrence to be comparable to, but slightly lower than, that previously reported in healthy human subjects (via both video tracking and search coil recordings [4]). Also in agreement with previous human studies, we found that SWJ saccades tended to be larger and faster than non-SWJ saccades, and that both SWJ and non-SWJ saccades fell on the same peak velocity-magnitude relationship main sequence (Fig 2). The likelihood of a given saccade to be part of a SWJ increased with saccadic magnitude (Fig 4C) and distance to the fixation target (Fig 4B), also consistent with previous findings for human SWJs. Return saccade latencies were shorter after large fixation errors (Fig 5), further supporting the notion that primate SWJs, similarly to human SWJs, help to correct fixation position during normal vision. These combined results suggest a similar coupling mechanism in human and primate SWJs, where large fixational saccades are usually followed by subsequent corrective saccades, thereby producing SWJs as a result.

Whereas fixational saccades tend to be horizontal in humans, and SWJs even more strongly so [4], fixational saccades in macaques do not typically show a horizontal preference [12,15]. Indeed, here we found fixational saccades to be predominantly vertical, both inside and outside primate SWJs (Fig 3 and S2 Fig). This dramatic distinction in the directional components of human and primate SWJs seems to make little difference to the coupling mechanisms linking the first and second SWJ saccades though, which appear to be fundamentally common to both species. Thus, our combined data suggest that fixational saccades (including those forming SWJs) have a common origin in primates and humans, even if the directional biases of monkey and human fixational saccades are grossly different.

These findings constrain the possible brain areas and mechanisms underlying microsaccade generation in both species. Premotor areas in the brain stem separate anatomically horizontal and vertical saccade generation: horizontal saccades are generated in the paramedian pontine reticular formation (pprf) and vertical saccades in the rostral interstitial nucleus of the medial longitudinal fasciculus (riMLF). Thus, there is no single brain stem area that can explain the generation of fixational saccades in both primates and humans. It could be that the pprf generates microsaccades in humans but not in monkeys, and that the riMLF generates microsaccades in monkeys but not humans—but there is no neurophysiological evidence to support this possibility.

Upstream, the superior colliculus (SC) encodes the vectors of saccades of all directions in a single 2D map, making this structure a good candidate for fixational saccade generation in human and nonhuman primates. This is consistent with recent evidence linking microsaccade generation to neural activity in the rostral SC [37] and showing decreased microsaccade rates after rostral SC inactivation [37,38]. Other potential areas that may provide a common vertical and horizontal microsaccade-triggering signal include the oculomotor areas in the cerebellum and the cortical frontal eye fields [39].

A recent study [38] has proposed that microsaccade generation results from an imbalance between the left and right superior colliculus. The fact that fixational saccades tended to be vertical in our primate population seems to indicate an alternative mechanism, given that a purely vertical fixational saccade would be accompanied by equivalent activity in the left and right SCs. Other generation models [40–42] have proposed more general mechanisms that trigger microsaccades when the overall pattern of activity in the SC changes, by increasing the activity level of a given point in the map, and/or changing the location of the center of mass of the population activity.

## Supporting Information

S1 Checklist The ARRIVE Guidelines Checklist. We followed the ARRIVE (Animal Research: Reporting of In Vivo Experiments) guidelines and the ARRIVE Checklist is available.
(DOC)

S1 Fig Normalized directions of SWJ saccades and non-SWJ saccades in each monkey. Despite individual differences across animals, there is a prominent vertical component for both SWJ saccades and non-SWJ saccades. This is in contrast to human SWJs, which are strongly horizontal.
(EPS)

S2 Fig Distribution of saccadic magnitudes for SWJ saccades and non-SWJ saccades in each monkey. (EPS)

S3 Fig Distribution of SWJ indices. Normalized distribution of SWJ indices in logarithmic scale. Dashed vertical line represents the index threshold used in the SWJ detection algorithm. Shadow indicates the s.e.m. across monkeys (*n* = 5).
(EPS)

S4 Fig Distribution of distances from the end of the second SWJ saccade to the fixation target, and from the end of the second SWJ saccade to the beginning of the first SWJ saccade. The normalized distributions from the end of the second SWJ saccade to the target, and from the end of the second SWJ saccade to the beginning of the first SWJ saccade did not differ significantly (Z-values = 1.75; *p* > 0.07). Shadows indicate the s.e.m. across monkeys (*n* = 5).
(EPS)
